# Impact of deubiquitination of Epstein-Barr virus Rta and Zta on lytic development

**DOI:** 10.1128/spectrum.04158-25

**Published:** 2026-05-21

**Authors:** Chi-Yuan Chen, Po-Chun Chen, Yi-Shan Lu, Xin-Ru Lin, Ya-Chun Yang, Yi-Kai Lee, Zi-Yun Cheng, Shu-Yin Chang, Ya-Fang Chiu, Li-Kwan Chang

**Affiliations:** 1 Department of Biochemical Science and Technology, College of Life Science, National Taiwan University, Taipei, Taiwan; 2 Chang Gung University, Graduate Institute of Biomedical Sciences 56081, Taoyuan, Taiwan; 3 Department of Microbiology and Immunology, Chang Gung University 56081, Taoyuan, Taiwan; 4 Research Center for Emerging Viral Infections, Chang Gung University 56081, Taoyuan, Taiwan; Emory University School of Medicine, Atlanta, Georgia, USA

**Keywords:** Epstein-Barr virus, Zta, Rta, RNF4, USP11

## Abstract

Rta and Zta are transcription factors expressed by Epstein-Barr virus (EBV) during the immediate-early stage of the lytic cycle. While these two proteins do not interact directly, they bind to a scaffold protein, RanBPM, to synergistically activate the transcription of EBV lytic genes. Earlier studies have shown that both Rta and Zta are destabilized through ubiquitination, which plays a crucial role in influencing EBV’s lytic development. It is also known that Rta’s ubiquitination is facilitated by the ubiquitin E3 ligase, RNF4. In this study, we show that RNF4 also promotes the ubiquitination of Zta. Meanwhile, a deubiquitinase, ubiquitin-specific protease (USP11), is known to interact with RanBPM. When tethered to RanBPM, Rta and Zta are deubiquitinated and stabilized by USP11. While host cells may utilize ubiquitination as a defense mechanism to destabilize Rta and Zta, thereby restricting EBV proliferation, EBV exploits RanBPM and the deubiquitination of Rta and Zta as a counter-defense strategy that favors EBV lytic replication. This study uncovers the interplay between the host and the viral mechanisms involving RNF4 and USP11 in modulating EBV lytic progression.

**IMPORTANCE:**

Epstein-Barr virus (EBV) is known to express two crucial transcription factors, Rta and Zta, which are essential for activating the transcription of viral lytic genes. These two proteins are indispensable for viral lytic proliferation. While Rta and Zta can function independently to promote transcription, they also cooperate synergistically, leading to robust expression of EBV lytic proteins. In an earlier study, we showed that this synergy requires the formation of a complex involving Rta, Zta, and RanBPM at the Zta response elements in EBV lytic promoters. Recent studies showed that, despite their importance, these transcription factors are destabilized by ubiquitination, potentially serving as a mechanism through which the host restricts EBV proliferation. This study shows that EBV exploits USP11, which interacts with RanBPM, to deubiquitinate and stabilize Rta and Zta. The stabilization by USP11 is critical for EBV to overcome host-imposed restrictions and maximize its lytic proliferation.

## INTRODUCTION

Epstein-Barr virus (EBV) is an oncogenic virus that infects human lymphoid and epithelial cells. While EBV is typically maintained in a latent state in B lymphocytes, the virus must enter a lytic phase to proliferate (1, 2). Rta and Zta, encoded by the EBV BRLF1 and BZLF1 genes, respectively, are transcription factors that transactivate EBV lytic genes (3–5). Rta and Zta bind to the Rta response element (RRE) and Zta response element (ZRE) in promoters, respectively, to activate transcription (4, 6–9). Notably, Zta uses this mechanism to enhance the transcription of the BRLF1 gene (10). In contrast, Rta forms a complex with MCAF1 and AP-1, which binds to the ZII region in the BZLF1 promoter to enhance BZLF1 transcription (11). Rta has also been identified as a major transactivator, capable of activating most of the viral early genes (12–14). Additionally, Rta interacts with MCAF1 to enhance Sp1-mediated transcription and its autoregulation (15, 16). Although Rta and Zta do not interact directly, both bind to MCAF1 or RanBPM, forming a complex on the ZRE, enabling Rta and Zta to cooperate synergistically in transactivating EBV lytic genes (17, 18).

Our earlier studies demonstrated that RNF4, also known as small nuclear RING finger protein, is a ubiquitin E3 ligase (19, 20) that promotes the ubiquitination of SUMO-2-conjugated Rta (21). RNF4 has been implicated in the lytic activation of EBV (21) and human adenovirus (22). RNF4 also facilitates the ubiquitination of SUMO-2-conjugated promyelocytic leukemia protein (PML) (23, 24), while a deubiquitinase, USP11, counteracts this activity to prevent PML degradation (25, 26). Furthermore, USP11 reduces ubiquitination to stabilize the E7 protein of human papillomavirus 16 (27) and the NP protein of influenza A virus (28, 29). USP11 also deubiquitinates p21 and increases its stability, thus inhibiting cell proliferation and tumorigenesis (30). Additionally, Zta is modified by SUMOs, with SUMOylation at the K12 residue inhibiting its transcriptional activity via association with the HDAC complex (31, 32). Zta has also been found to undergo ubiquitination, primarily at the K12 residue, along with several minor ubiquitination sites, including K188, K207, and K219 (33). However, the specific ubiquitin E3 ligases responsible for promoting Zta’s ubiquitination have yet to be identified.

RanBPM, a scaffold multifunctional protein, is known to regulate signaling pathways and plays a crucial role in influencing cellular functions (34, 35). Our earlier study showed that Rta and Zta simultaneously interact with RanBPM, enabling Rta and Zta to synergistically transactivate EBV lytic genes (18). Furthermore, RanBPM interacts with USP11, which removes ubiquitin from RanBPM, thereby stabilizing the protein (36). A recent study revealed that the RanBPM-USP11 axis regulates the stability of p21, thus affecting the DNA damage response (37). In this study, we find that RNF4 facilitates the ubiquitination of Zta. Our earlier study showed that RNF4 also facilitates the ubiquitination of Rta (21), indicating that RNF4 promotes the ubiquitination of both EBV immediate-early transcription factors. After binding to RanBPM, ubiquitinated Rta (Ub-Rta) and Zta are deubiquitinated by USP11, leading to the stabilization of these two proteins and an increase in the transactivation of EBV lytic genes. Additionally, we show that knocking down USP11 expression attenuates EBV lytic progression, highlighting the importance of USP11 in supporting EBV lytic activation.

## RESULTS

### RNF4 interacts with Zta

**Fig 1:**
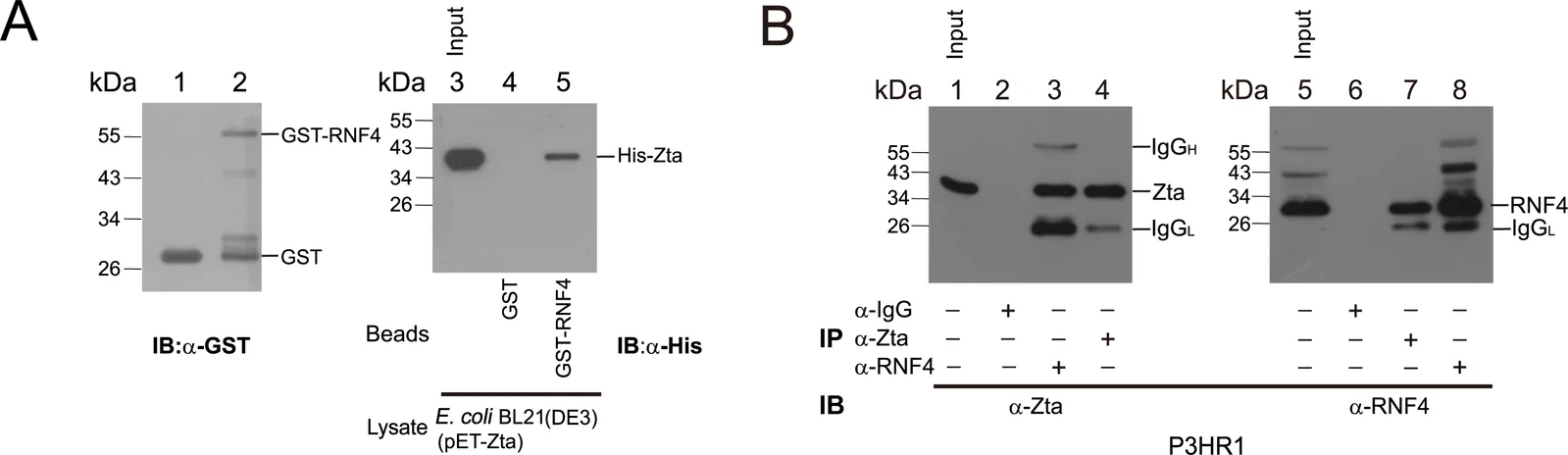
Interaction between Zta and RNF4. (**A**) GST-glutathione-Sepharose beads (lane 1) or GST-RNF4-glutathione-Sepharose beads (lane 2) were added to the lysate from *E. coli* BL21(DE3) (pET-Zta). His-Zta captured by GST-glutathione-Sepharose (lane 4) and GST-RNF4-glutathione-Sepharose (lane 5) beads was detected by immunoblotting (IB) using anti-His (lanes 3–5) antibody. Lane 3 was loaded with 1% of the lysate. (**B**) P3HR1 cells were treated with TPA and sodium butyrate for 24 h to induce the EBV lytic cycle and activate the expression of Zta. Proteins in the lysates were immunoprecipitated (IP) with anti-Zta (lanes 4 and 7), anti-RNF4 (lanes 3 and 8), or anti-IgG (lanes 2 and 6) antibodies, and then detected by immunoblotting with anti-Zta (lanes 1–4) and anti-RNF4 (lanes 5–8) antibodies. Input lanes (lanes 1 and 5) were loaded with 1% of the lysates. IgG_L_ and IgG_H_: light and heavy chains of IgG, respectively.

In an earlier study, we demonstrated that RNF4 functions as a ubiquitination E3 ligase for Rta (21). During that investigation, we found evidence suggesting that RNF4 may also interact with Zta (20). To further explore this possibility, we conducted a GST-pulldown assay to verify the interaction between Zta and RNF4. We confirmed the presence of GST and GST-RNF4 in the lysate from *Escherichia coli* BL21(DE3) (pGEX-4T1) and *E. coli* BL21(DE3) (pGEX-RNF4), respectively (Fig. 1A, lanes 1 and 2). Glutathione-Sepharose beads were then added to these lysates to facilitate the binding of GST and GST-RNF4 to the beads. After washing, the beads were mixed with the lysate from *E. coli* BL21(DE3) (pET-Zta). We detected His-Zta in the lysate (Fig. 1A, lane 3) and observed that His-Zta was pulled down by GST-RNF4-glutathione-Sepharose beads but not GST-glutathione-Sepharose beads (Fig. 1A, lanes 4 and 5), indicating a direct interaction between RNF4 and Zta *in vitro*. To confirm this interaction *in vivo*, P3HR1 cells were treated with sodium butyrate and 12-*O*-tetradecanoylphorbol-13-acetate (TPA) to activate the EBV lytic cycle and Zta expression. A cell lysate was prepared at 24 h post-activation. Immunoblot analysis using anti-Zta and anti-RNF4 antibodies revealed the presence of both Zta and RNF4 in the lysate (Fig. 1B, lanes 1 and 5). We found that adding the anti-Zta antibody to the lysate immunoprecipitated Zta (Fig. 1B, lane 4) and also coimmunoprecipitated RNF4 (Fig. 1B, lane 7). Additionally, the anti-RNF4 antibody immunoprecipitated RNF4 (Fig. 1B, lane 8) and coimmunoprecipitated Zta (Fig. 1B, lane 3). A parallel experiment showed that neither Zta nor RNF4 was immunoprecipitated by an anti-IgG antibody (Fig. 1B, lanes 2 and 6). Together, these results indicated that Zta interacts with RNF4 *in vivo*.

### RNF4 promotes Zta’s ubiquitination

**Fig 2:**
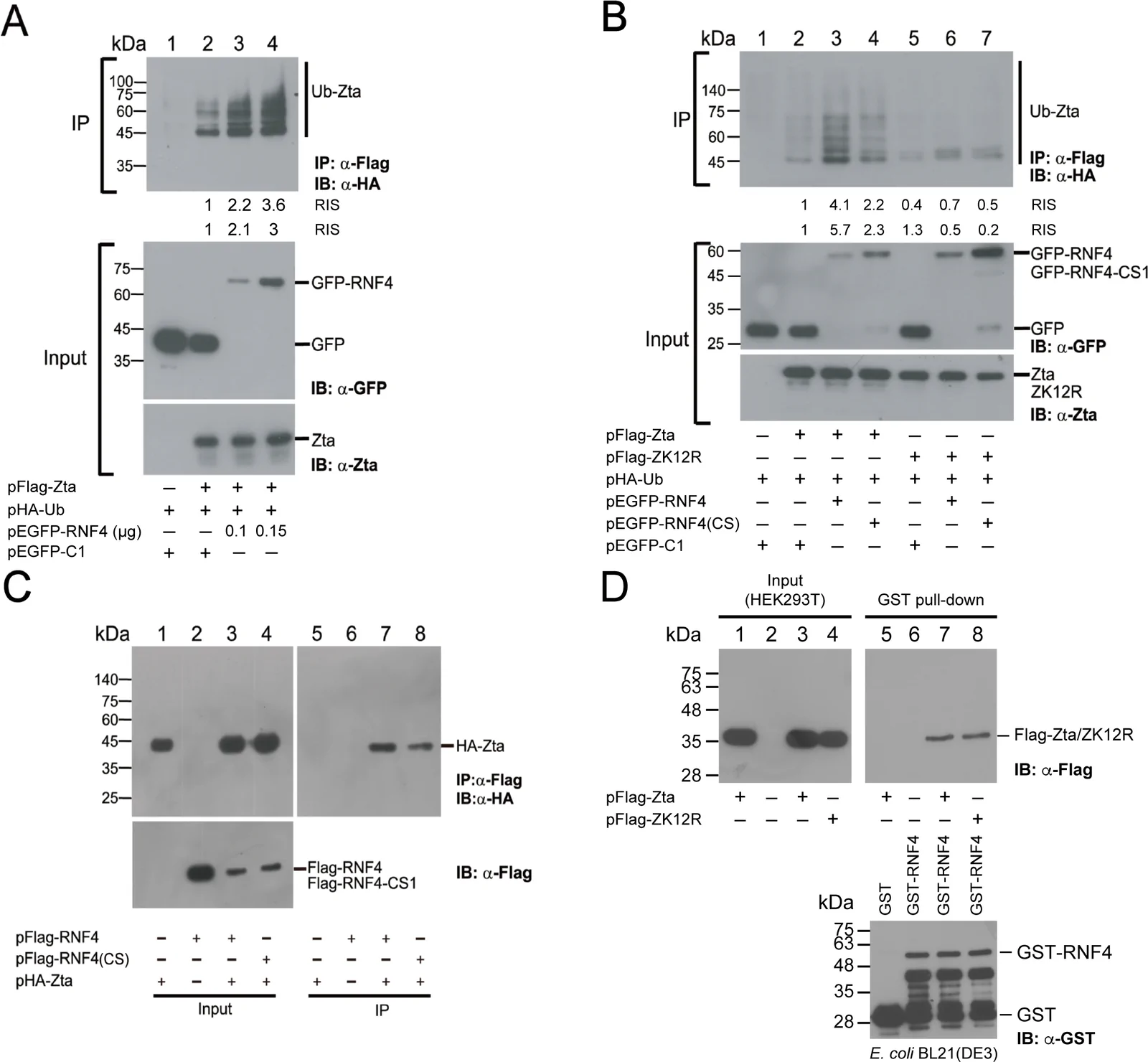
Promotion of Zta’s ubiquitination by RNF4. HEK293T cells were cotransfected with (**A**) pFlag-Zta, pHA-Ub, and pEGFP-RNF4, or (**B**) with pHA-Ub, pEGFP-RNF4, or pEGFP-RNF4(CS), and pFlag-Zta or pFlag-ZK12R. At 24 h after transfection, cells were treated with MG132 for 12 h. Proteins in the lysate were immunoprecipitated (IP) using Flag-M2 agarose beads; proteins bound to the beads were analyzed by immunoblotting (IB) with anti-HA antibody. Input lanes were loaded with 6% of the lysate for anti-GFP antibody; 1% for anti-Zta antibody. (**C**) Cells were cotransfected with pHA-Zta and pFlag-RNF4 or pFlag-RNF4(CS). Proteins in the lysate were immunoprecipitated with anti-Flag M2 agarose beads and detected by immunoblotting with anti-HA antibody. (**D**) *E. coli* BL21(DE3) expressed GST and GST-RNF4 were bound to glutathione-Sepharose beads. The beads were then mixed with cell lysate from HEK293T cells transfected with pFlag-Zta or pFlag-ZK12R. Proteins bound to beads were analyzed by immunoblotting with anti-Flag antibody. Input lanes were loaded with 1% of the lysate for anti-Flag antibody and anti-GST antibody. The relative intensity of smear (RIS) quantifies the intensity distribution within a smear, measured using ImageJ software. The top row displays the RIS corresponding to the smear shown, while the bottom row presents the RIS from a different experiment not depicted in the figure.

After demonstrating the interaction between Zta and RNF4, we further examined whether RNF4 promotes Zta’s ubiquitination. HEK293T cells were cotransfected with pFlag-Zta, pHA-Ub, and an empty vector, pEGFP-C1. Denatured immunoprecipitation using an anti-Flag antibody, followed by detection via immunoblot analysis with an anti-HA antibody, revealed a series of bands larger than the expected size of Zta (38 kDa; Fig. 2A, lane 2); these bands were undetectable in lysates from cells that were not transfected with pFlag-Zta (Fig. 2A, lane 1), suggesting they represent ubiquitinated Zta (Ub-Zta).

We also cotransfected HEK293T cells with pFlag-Zta, pHA-Ub, and either pEGFP-RNF4 or an empty vector, pEGFP-C1, and found that transfecting 0.1 and 0.15 µg pEGFP-RNF4 increased the intensity of the bands corresponding to ubiquitinated Zta in a dose-dependent manner (Fig. 2A, lanes 3 and 4). A similar transfection experiment was performed using an RNF4 mutant, GFP-RNF4(CS), which has mutations in the RING domain and lacks ubiquitin E3 ligase activity (21). The CS mutation significantly reduced the ability of GFP-RNF4 to promote the ubiquitination of Zta (Fig. 2B, lanes 3 and 4), confirming that RNF4 promotes Zta’s ubiquitination.

Zta is predominantly ubiquitinated at K12 (33); thus, we constructed pFlag-ZK12R, which encodes a Zta mutant where the K12 residue is substituted with arginine (ZK12R). After cotransfecting HEK293T cells with pFlag-ZK12R, pHA-Ub, and pEGFP-RNF4 or pEGFP-RNF4(CS), we prepared lysates at 24 h after transfection. Immunoprecipitation indicated that ubiquitination of Flag-ZK12R occurred at a substantially lower level compared to Flag-Zta (Fig. 2B, lanes 2 and 5). Furthermore, neither GFP-RNF4 nor RNF4(CS) could enhance the ubiquitination of ZK12R (Fig. 2B, lanes 6 and 7). Notably, a low level of ubiquitination of ZK12R was observed (Fig. 2B, lanes 5–7), suggesting minor ubiquitination at three alternative sites: K188, K207, or K219, as previously indicated (33). To confirm that the decrease in Zta ubiquitination was not due to a loss of interaction between Zta and RNF4(CS) arising from the CS mutations, we cotransfected HEK293T cells with plasmids expressing HA-Zta, and either Flag-RNF4 or Flag-RNF4(CS). Immunoprecipitation with an anti-Flag antibody followed by immunoblotting with an anti-HA antibody showed that HA-Zta was coimmunoprecipitated with Flag-RNF4 and Flag-RNF4(CS) by the anti-Flag antibody (Fig. 2C, lanes 7 and 8), indicating that Zta interacts with both variants of RNF4. Thus, the observed decrease in Zta ubiquitination by GFP-RNF4(CS) was not due to a lack of interaction between the proteins.

Additionally, we conducted a GST-pulldown assay to investigate the interaction between RNF4 and Zta or ZK12R. Proteins from lysates of HEK293T(pFlag-Zta) and HEK293T(pFlag-ZK12R) cells were mixed with bacterially expressed GST-RNF4 bound to glutathione-Sepharose beads. Immunoblotting revealed that Zta in the lysate (Fig. 2D, lanes 1 and 3) was pulled down by GST-RNF4-glutathione-Sepharose beads (Fig. 2D, lane 7) but not by GST-glutathione-Sepharose beads (Fig. 2D, lane 5). ZK12R in the lysate (Fig. 2D, lane 4) was also pulled down by GST-RNF4-glutathione-Sepharose beads (Fig. 2D, lane 8), demonstrating that both Zta and ZK12R interact with RNF4. The results indicate that RNF4 promotes the ubiquitination of Zta.

### Destabilization of Zta by RNF4

**Fig 3:**
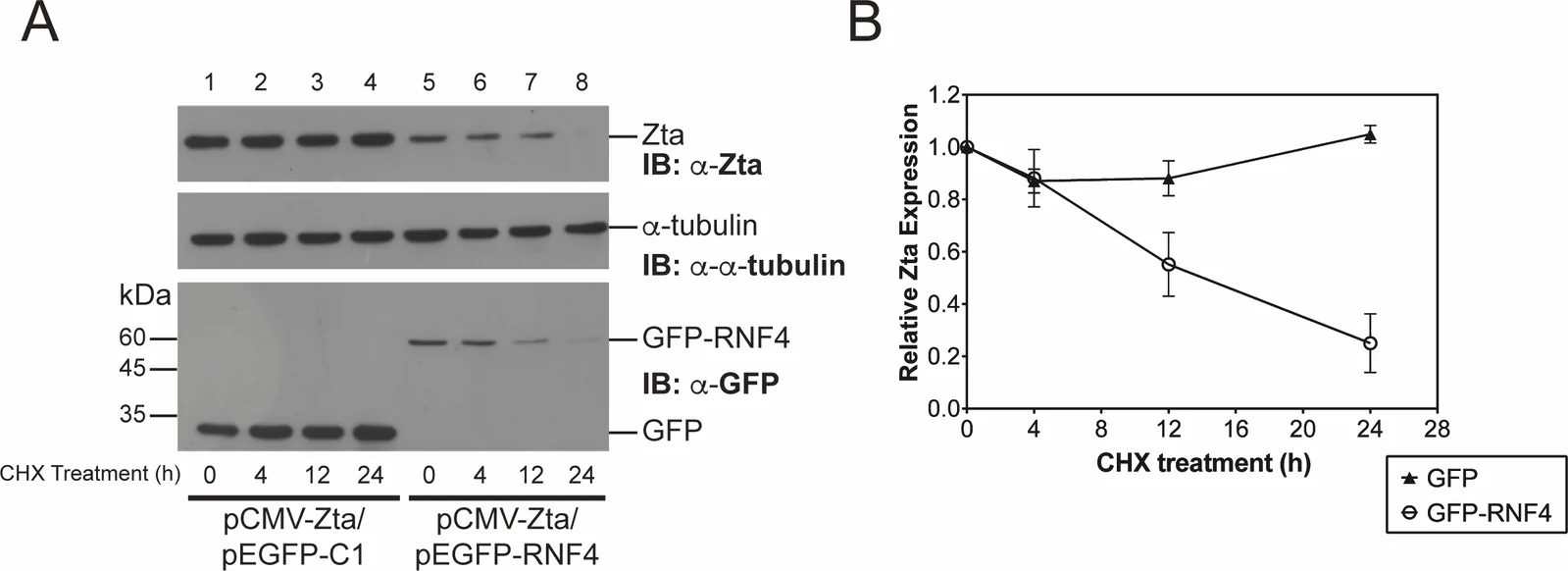
Influence of RNF4 on the stability of Zta. (**A**) HEK293T cells were cotransfected with pCMV-Zta and either pEGFP-C1 (lanes 1–4) or pEGFP-RNF4 (lanes 5–8). At 40 h posttransfection, cells were treated with 300 μM cycloheximide (CHX). At 0 h, 4 h, 12 h, and 24 h after CHX treatment, cells were lysed, and proteins in the lysate were analyzed by immunoblotting (IB) with (**A**) anti-Zta, anti-GFP, and anti-α-tubulin antibodies. (**B**) The intensity of the Zta bands in panel **A** was measured using ImageJ software and presented in a line chart from three independent experiments. The intensity of the Zta band was normalized to the intensity of α-tubulin. The intensity of the Zta band at 0 h of CHX treatment was set to 100%. The experiment was repeated three times. Bar: standard deviation.

An earlier study demonstrated that ubiquitination destabilizes Zta (33). Given that our findings indicate that RNF4 promotes Zta’s ubiquitination (Fig. 2), we hypothesize that expressing RNF4 would likely destabilize Zta. To test this, we cotransfected HEK293T cells with pCMV-Zta and an empty vector, pEGFP-C1. At 40 h post-transfection, cells were treated with cycloheximide (CHX) to inhibit protein synthesis. Immunoblot analysis revealed that Zta remained stable, with levels relatively constant after CHX treatment over a 24 h period (Fig. 3A, lanes 1–4). However, when cells were cotransfected with pCMV-Zta and pEGFP-RNF4, a decrease in Zta levels was observed even at 0 h post-CHX treatment (Fig. 3A, lanes 5–8). Specifically, Zta levels were reduced by approximately 12% and 45% at 4 h and 12 h after CHX treatment, respectively, and further declined to less than 25% at 24 h (Fig. 3B). The half-life was estimated to be 16 h (Fig. 3B), indicating that ubiquitination by RNF4 reduces Zta stability.

### USP11 deubiquitinates Zta

**Fig 4:**
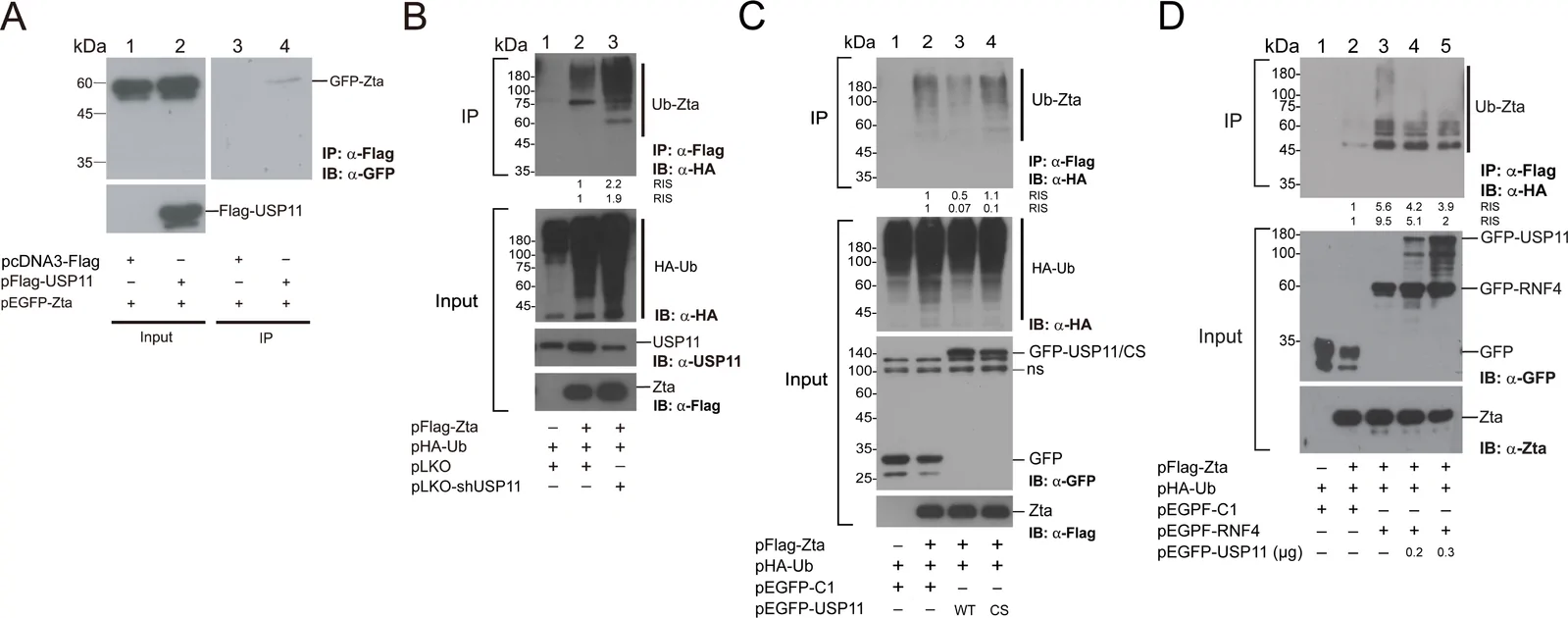
Deubiquitination of Zta by USP11. HEK293T cells were cotransfected with pFlag-USP11 and pEGFP-Zta (lanes 2 and 4) or pcDNA3-Flag and pEGFP-Zta (lanes 1 and 3) (**A**); pFlag-Zta and pHA-Ub, and pLKO or pLKO-shUSP11 (**B**); pHA-Ub and pEGFP-C2; pFlag-Zta, pHA-Ub, and pEGFP-C2; pFlag-Zta, pHA-Ub, and pEGFP-USP11 (WT) or pEGFP-USP11(CS) (CS) (**C**); pFlag-Zta, pHA-Ub, and pEGFP-RNF4 or pEGFP-USP11 (**D**). At 24 h after transfection, a cell lysate was prepared, and proteins in the lysates were immunoprecipitated (IP) with anti-Flag antibody and detected by immunoblotting (IB) with anti-GFP antibody (lanes 3 and 4) (**A**). At 24–48 h after transfection, cells were treated with 2.5 µM MG132 for another 12 h. Proteins in the lysate were then immunoprecipitated with anti-Flag antibody and detected by immunoblotting with anti-HA antibody (**B–D**). Input lanes show the GFP-fusion proteins in 6% of the lysate, which were detected by immunoblotting using the anti-GFP antibody. Input lanes were loaded with 1% of the lysate and immunoblotted with anti-Flag antibody and anti-HA antibody. The relative intensity of smear (RIS) quantifies the intensity distribution within a smear, measured using ImageJ software. The top row displays the RIS corresponding to the smear shown, while the bottom row presents the RIS from a different experiment not depicted in the figure. “ns”: a band nonspecifically detected by immunoblotting.

An earlier study demonstrated that USP11 interacts with and deubiquitinates RanBPM (36). Our previous work indicated that Zta also interacts with RanBPM (18). Given that both USP11 and Zta interact with RanBPM, this study aimed to investigate whether USP11 deubiquitinated Zta. We cotransfected HEK293T cells with plasmids expressing GFP-Zta and Flag-USP11 (Fig. 4A). At 24 h post-transfection, proteins in the lysate were immunoprecipitated using an anti-Flag antibody. Immunoblotting with an anti-GFP antibody demonstrated that GFP-Zta was coimmunoprecipitated with Flag-USP11 (Fig. 4A, lane 4). Conversely, GFP-Zta was not immunoprecipitated by the anti-Flag antibody when the cells were cotransfected with pEGFP-Zta and an empty vector, pcDNA3-Flag (Fig. 4A, lane 3), confirming that GFP-Zta interacts with Flag-USP11. To further investigate the functional relationship between Zta and USP11, we cotransfected HEK293T cells with plasmids expressing Flag-Zta, HA-Ub, and either pLKO-shUSP11, which expresses USP11 shRNA, or pLKO. At 48 h post-transfection, proteins immunoprecipitated with the anti-Flag antibody were analyzed via immunoblotting with the anti-HA antibody. We observed that Flag-Zta was ubiquitinated when the cells were cotransfected with pFlag-Zta, pHA-Ub, and pLKO (Fig. 4B, lane 2). In contrast, cotransfection with pFlag-Zta, pHA-Ub, and pLKO-shUSP11 resulted in a reduced level of endogenous USP11 (Fig. 4B, lanes 2 and 3), demonstrating effective knockdown of USP11 by shRNA. Notably, this reduction in USP11 expression led to an increase in the level of ubiquitinated Zta (Fig. 4B, lane 3). It is known that cysteine-to-serine substitutions at C275 and C283 in USP11, termed USP11(CS), abolish its deubiquitination activity (27). In another cotransfection experiment, we showed that co-expressing Flag-Zta and GFP-USP11 in HEK293T cells led to a decrease in the level of ubiquitinated Zta (Fig. 4C, lane 3). However, this decrease was unobserved when cells were cotransfected with plasmids expressing Flag-Zta and GFP-USP11(CS) (Fig. 4C, lane 4), indicating that the mutation impairs USP11’s ability to deubiquitinate Zta. Finally, we cotransfected the cells with pFlag-Zta, pHA-Ub, and either the empty vector, pEGFP-C1, or pEGFP-RNF4. As anticipated, transfection with pEGFP-RNF4 increased Zta ubiquitination levels (Fig. 4D, lane 3). Conversely, cotransfection with 0.2 µg or 0.3 µg of pEGFP-USP11 reduced the levels of ubiquitinated Zta (Fig. 4D, lanes 4 and 5), further demonstrating that Zta is deubiquitinated by USP11.

### Stabilization of Zta by USP11

**Fig 5:**
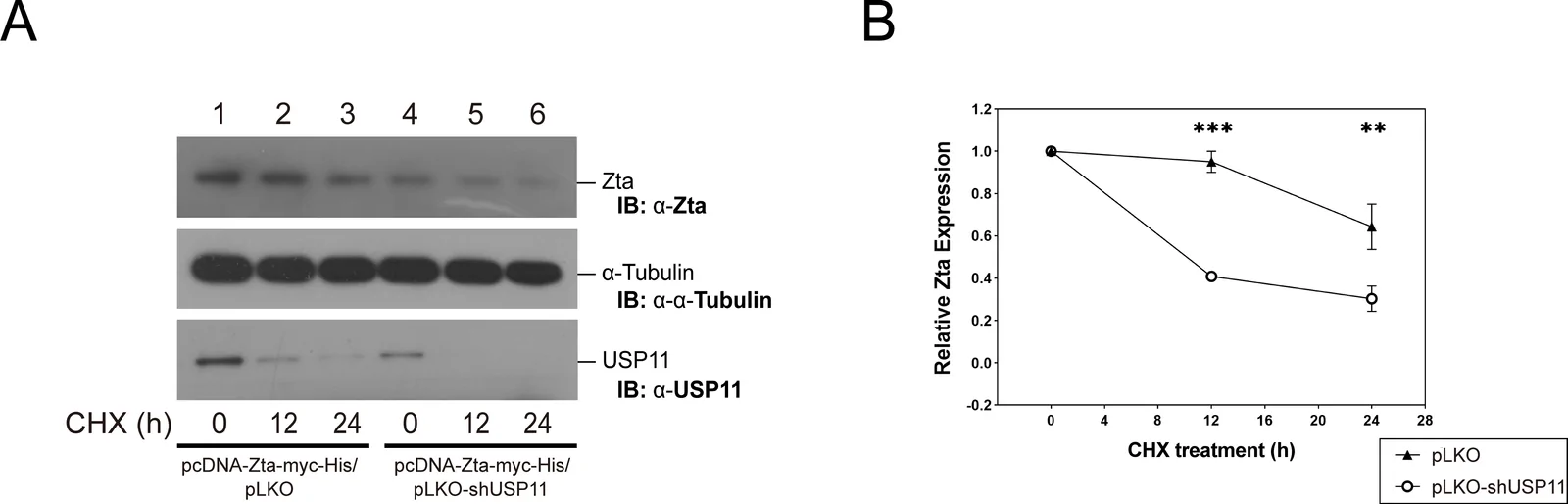
Influence of USP11 on the stability of Zta. (**A**) HEK293T cells were cotransfected with pcDNA-Zta-myc-His and pLKO (Ctrl-shRNA; lanes 1–3) or pLKO-shUSP11 (USP11-shRNA; lanes 4–6). At 40 h post-transfection, cells were treated with 200 μM cycloheximide (CHX). At 0 h, 12 h, and 24 h after CHX treatment, cells were lysed, and proteins in the lysate were analyzed by immunoblotting (IB) with (**A**) anti-Zta, anti-USP11, and anti-α-tubulin antibodies. The intensity of the Zta band in panel **A** was determined using ImageJ software and presented in a line chart from three independent experiments. The intensity of the Zta band at 0 h of CHX treatment was set to 100% (**B**). Data are presented as mean with standard deviation from three independent experiments. The results were analyzed statistically with Student’s *t*-test. **, *P* < 0.01; ***, *P* < 0.001.

HEK293T cells were cotransfected with pcDNA-Zta-myc-His and either pLKO-shUSP11 or pLKO to examine the role of USP11 in stabilizing Zta. The results indicated that the expression of control shRNA slightly reduced the amounts of Zta by 12 h after CHX treatment (Fig. 5A and B). In contrast, a 60% reduction in Zta levels was observed at 12 h when the cells were transfected with USP11 shRNA (Fig. 5B). By 24 h post-CHX treatment, control shRNA reduced Zta abundance by 35%, while cells expressing USP11 shRNA exhibited a 65% reduction in Zta levels (Fig. 5B). These findings indicated that the reduction of USP11 destabilizes Zta.

### USP11 deubiquitinates Rta

**Fig 6:**
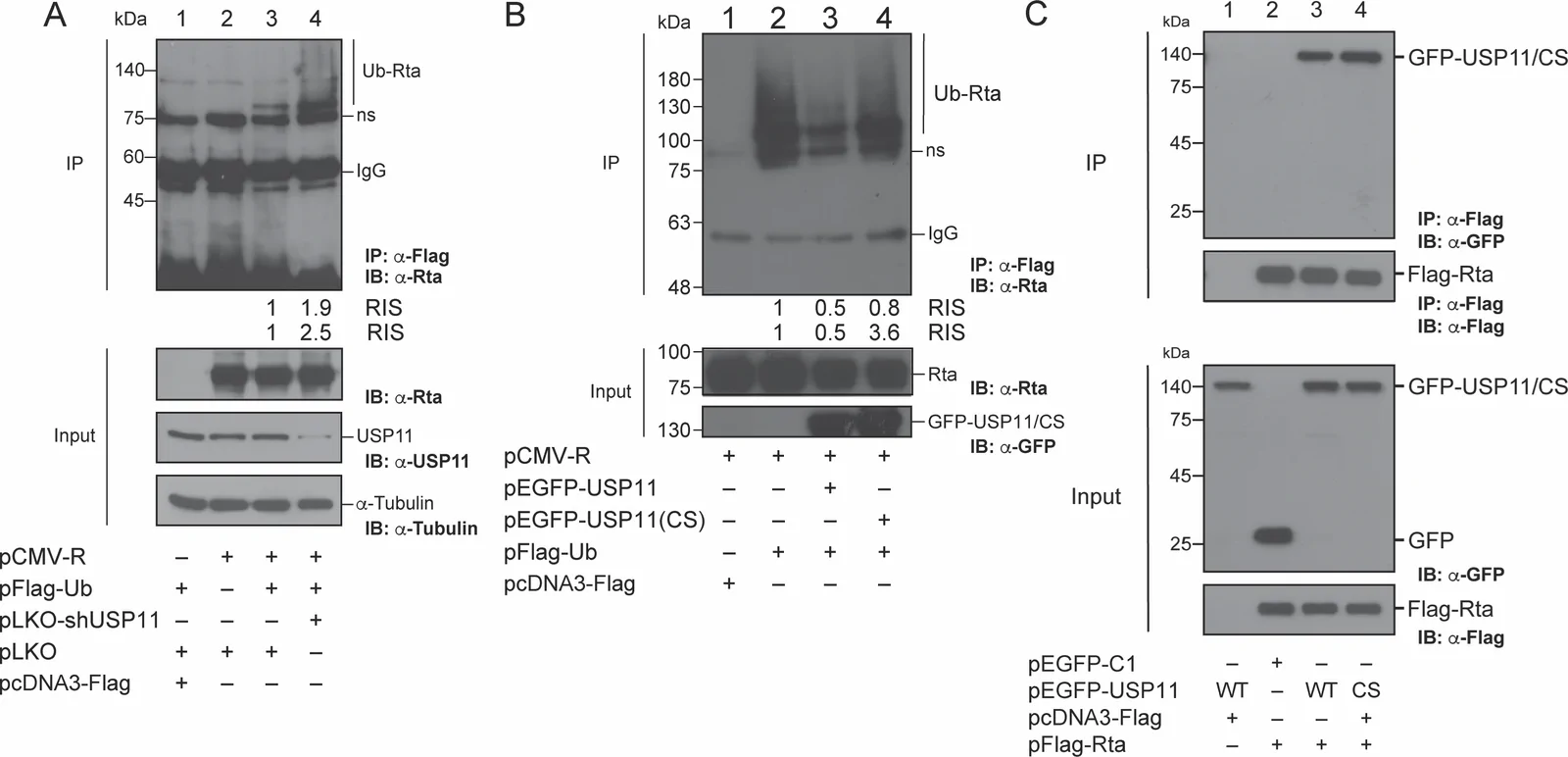
Deubiquitination of Rta by USP11. (**A**) HEK293T cells were cotransfected with pCMV-R, pFlag-Ub, and pLKO-shUSP11 or pLKO, which expresses control shRNA. At 48 h after transfection, cells were treated with 5 µM MG132 for 8 h to accumulate polyubiquitinated proteins. Subsequently, proteins in the lysate were immunoprecipitated (IP) with anti-Flag M2 agarose. Thereafter, immunoprecipitated polyubiquitinated Rta (Ub-Rta) was analyzed by immunoblotting (IB) using an anti-Rta antibody. Proteins in the lysate were detected by immunoblotting with anti-Rta, anti-USP11, and anti-α-tubulin antibodies (input). (**B**) A similar experiment in panel **A** was conducted except that HEK293T cells were cotransfected with pCMV-R, pFlag-Ub, and pEGFP-USP11 or pEGFP-USP11-CS. (**C**) HEK293T cells were cotransfected with plasmids encoding Flag-Rta and GFP-USP11 or GFP-USP11-CS. Proteins in the lysate were immunoprecipitated with an anti-Flag antibody and detected by immunoblotting with an anti-GFP antibody to determine the interaction of Rta with USP11 or USP11(CS). Proteins in the lysate were also detected by immunoblotting with anti-GFP and anti-Flag antibodies (input). Input lanes were loaded with 1% of the lysate for detection by anti-Rta, anti-USP11, and anti-α-tubulin antibodies; 5% of the lysate for detection by an anti-GFP antibody. The relative intensity of smear (RIS) quantifies the intensity distribution within a smear, measured using ImageJ software. The top row displays the RIS corresponding to the smear shown, while the bottom row presents the RIS from a different experiment not depicted in the figure.

Our earlier studies demonstrated that Rta interacts with RanBPM and is ubiquitinated by RNF4 (21, 38); this study investigated whether Rta, similar to Zta, was deubiquitinated by USP11. To test this, HEK293T cells were cotransfected with pCMV-R, pFlag-Ub, and either pLKO-shUSP11 or pLKO. At 48 h after transfection, proteins in the lysates were immunoprecipitated using anti-Flag M2 agarose beads. The immunoprecipitated Ub-Rta was detected by immunoblotting with an anti-Rta antibody. The results showed that Ub-Rta bands were undetectable when the cells were cotransfected with pLKO and pCMV-R or pFlag-Ub (Fig. 6A, lanes 1 and 2). However, Ub-Rta bands were present when the cells were cotransfected with pCMV-R, pFlag-Ub, and pLKO (Fig. 6A, lane 3). Furthermore, the inhibition of USP11 expression by shRNA resulted in increased levels of ubiquitinated Rta (Fig. 6A, lane 4). A similar experiment was conducted using HEK293T cells cotransfected with pCMV-Rta, pFlag-Ub, and either pEGFP-USP11 or pEGFP-USP11(CS) (Fig. 6B, lanes 3 and 4). At 24 h after transfection, cell lysates were prepared, and proteins in the lysate were immunoprecipitated with anti-Flag M2 agarose beads. The results indicated that the level of ubiquitinated Rta decreased following the expression of GFP-USP11 (Fig. 6B, lane 3), while only a little reduction was observed with GFP-USP11(CS) (Fig. 6B, lane 4). Collectively, these findings demonstrate that ubiquitinated Rta is deubiquitinated by USP11. The study also examined whether Rta interacted with GFP-USP11 and whether the CS mutation affected this interaction. HEK293T cells were transfected with pFlag-Rta and either pEGFP-USP11 or pEGFP-USP11(CS). Proteins in the lysate were immunoprecipitated with an anti-Flag antibody and detected by immunoblotting with an anti-GFP antibody. Analysis revealed that Rta was coimmunoprecipitated with GFP-USP11 or GFP-USP11(CS) (Fig. 6C, lanes 3 and 4), indicating that the lack of deubiquitination of Flag-Rta was not due to a mutation that prevents the interaction between USP11(CS) and Flag-Rta.

### Influence of USP11 on the stability of Rta

**Fig 7:**
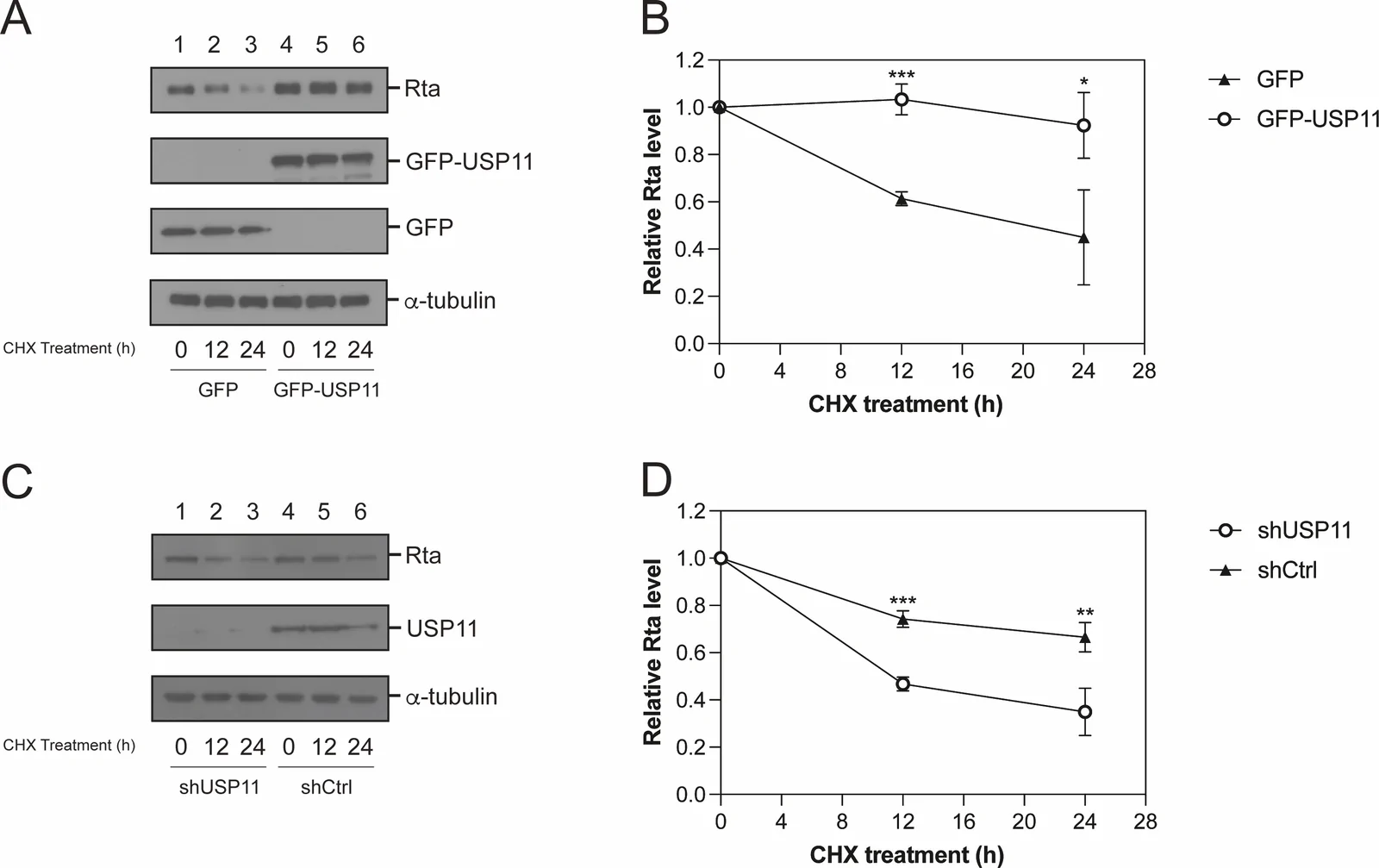
Stabilization of Rta by USP11. (**A**) HEK293T cells were transfected with plasmids expressing Rta, GFP-USP11 (lanes 4–6) or GFP (lanes 1–3). At 24 h after transfection, cells were treated with 50 µg/mL cycloheximide (CHX) to inhibit protein synthesis. A cell lysate was prepared at 0 h, 12 h, and 24 h after CHX treatment, and proteins in the lysate were detected by immunoblotting using the indicated antibodies. (**C**) A similar experiment in panel **A** was performed except that pEGFP-USP11 was replaced with pLKO-shUSP11 (USP11 shRNA; shUSP11; lanes 1–3) or pLKO (control shRNA; shCtrl; lanes 4–6). (**B and D**) A densitometric analysis of Rta level normalized to α-tubulin was performed using ImageJ software. Data are presented as mean with standard deviation from three independent experiments. The results were analyzed statistically with Student’s *t*-test. *, *P* < 0.05; **, *P* < 0.01; ***, *P* < 0.001.

To investigate whether the expression of USP11 affects the stability of Rta, HEK293T cells were cotransfected with pCMV-R and either pEGFP-USP11 (GFP-USP11) or pEGFP-C1 (GFP). At 24 h after transfection, cells were treated with CHX. At 12 h after the treatment, Rta levels decreased by 40% when cells were cotransfected with pCMV-R and pEGFP-C1 (Fig. 7A, lanes 1 and 2; 7B). In contrast, a reduction of Rta was unobserved if the cells were cotransfected with pCMV-R and pEGFP-USP11 (Fig. 7A, lanes 4 and 5; 7B). At 24 h after CHX treatment, a 60% reduction in Rta expression was noted in cells cotransfected with pCMV-Rta and pEGFP-C1 (Fig. 7A, lanes 1 and 3). Only a slight decrease was observed in cells with pCMV-Rta and pEGFP-USP11 (Fig. 7A, lanes 4 and 6). A similar experiment was performed using HEK293T cells cotransfected with pCMV-R and either pLKO-shUSP11 or pLKO. The results indicated that reducing USP11 expression by shRNA led to a decrease in Rta levels by 52% at 12 h and 64% at 24 h after CHX treatment (Fig. 7C, lanes 1–3; 7D). Additionally, a reduction of Rta was also observed with control shRNA, with levels decreasing by 31% at 24 h (Fig. 7C, lanes 4 and 6; 7D). Taken together, these results demonstrate that USP11 stabilizes Rta.

### Involvement of USP11 and RanBPM in deubiquitination of Rta and Zta

**Fig 8:**
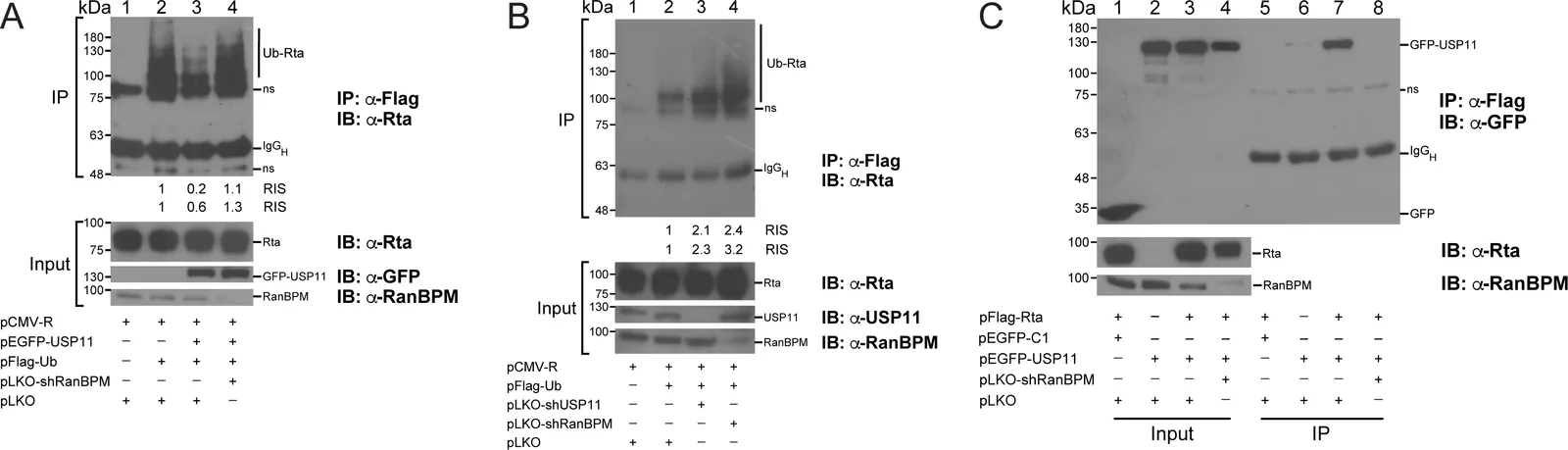
Involvement of RanBPM in deubiquitination of Rta by USP11. (**A**) HEK293T cells were cotransfected with pCMV-R, pFlag-Ub, pEGFP-USP11, and pLKO-shRanBPM or pLKO. The cells were treated with 5 µM MG132 to inhibit proteasomal degradation of ubiquitinated Rta. At 48 h after transfection, proteins in the lysate were immunoprecipitated (IP) using anti-Flag M2 agarose beads. Immunoprecipitated proteins were then immunoblotted (IB) with an anti-Rta antibody to detect ubiquitinated Rta. Proteins in the lysate were also detected by immunoblotting with anti-Rta, anti-GFP, and anti-RanBPM antibodies (input). (**B**) HEK293T cells were cotransfected with pCMV-R, pFlag-Ub, and pLKO-shUSP11, pLKO-shRanBPM, or pLKO. Ubiquitinated Rta was immunoprecipitated using an anti-Flag antibody and detected by immunoblotting with the anti-Rta antibody. (**C**) HEK293T cells were cotransfected with plasmids encoding Flag-Rta, GFP-USP11, RanBPM shRNA, or control shRNA. At 48 h after transfection, proteins in the lysate were coimmunoprecipitated by an anti-Flag antibody and detected by immunoblotting using an anti-GFP antibody. Proteins in the lysate were detected by immunoblotting with anti-Rta and anti-RanBPM antibodies. Input lanes were loaded with 1% of the lysate for detection by the anti-Rta antibody; 5% for detection by anti-GFP, anti-USP11, and anti-RanBPM antibodies. IgG_H_: heavy chain of IgG. ns: a band nonspecifically detected by immunoblotting. The relative intensity of smear (RIS) quantifies the intensity distribution within a smear, measured using ImageJ software. The top row displays the RIS corresponding to the smear shown, while the bottom row presents the RIS from a different experiment not depicted in the figure.

RanBPM is a scaffold protein that interacts with Rta, Zta, and USP11 (18, 35, 36, 38). The interaction between Rta and Zta with RanBPM facilitates their cooperative activation of EBV lytic transcription (18). This study aimed to determine whether RanBPM influences the deubiquitination of Rta and Zta by USP11. To demonstrate that USP11 deubiquitinates Rta, HEK293T cells were cotransfected with pCMV-R and pFlag-Ub. At 24 h after cotransfection, proteins in the lysate were immunoprecipitated and subsequently immunoblotted using anti-Flag M2 agarose beads and an anti-Rta antibody, respectively (Fig. 8A, lane 2). As expected, polyubiquitinated Rta bands were observed; however, these bands were undetected in the lysate from cells transfected with only pFlag-Ub (Fig. 8A, lane 1). Notably, a nonspecific protein band was detected at about 80 kDa, which is close to the location where Rta migrates on the gel (Fig. 8A, lane 1). The nature of this protein is unknown, but it is consistently detected when using an anti-Rta antibody to immunoblot the proteins that had been immunoprecipitated with an anti-Flag antibody (21). Additionally, the amount of polyubiquitinated Rta was found to decrease when the cells were cotransfected with pCMV-R, pFlag-Ub, and pEGFP-USP11 (Fig. 8A, lane 3), suggesting that the expression of GFP-USP11 reduces the amount of Ub-Rta. When cells were cotransfected with a plasmid expressing RanBPM shRNA, which reduced endogenous RanBPM expression, an increase in levels of ubiquitinated Rta was observed (Fig. 8A, lane 4), indicating that RanBPM is crucial for the deubiquitination of Rta by USP11.

To further investigate how USP11 and RanBPM influence Rta’s ubiquitination, the study employed USP11 shRNA and RanBPM shRNA, which effectively inhibited the expression of USP11 and RanBPM (Fig. 8B, lanes 3 and 4). HEK293T cells were cotransfected with plasmids expressing Rta, Flag-Ub, and USP11 shRNA (Fig. 8B, lane 3). The result showed an increase in the levels of polyubiquitinated Rta upon introducing USP11 shRNA (Fig. 8B, lanes 2 and 3). The study investigated the role of RanBPM in the ubiquitination and interaction of Rta with USP11. Upon cotransfection of HEK293T cells with plasmids expressing Rta, Flag-Ub, and RanBPM shRNA, an increase in polyubiquitinated Rta levels was observed (Fig. 8B, lanes 2 and 4).

Furthermore, coimmunoprecipitation assays of HEK293T cells that had been cotransfected with plasmids expressing Flag-Rta and GFP-USP11 demonstrated that in the absence of RanBPM shRNA, GFP-USP11 coimmunoprecipitated with Flag-Rta (Fig. 8C, lane 7). Conversely, when RanBPM shRNA was introduced, the interaction between GFP-USP11 and Flag-Rta was substantially reduced (Fig. 8C, lane 8), indicating that RanBPM is essential for the interaction between Rta and USP11. These findings suggest that RanBPM plays a crucial role in facilitating the deubiquitination and stabilization of Rta by USP11.

In additional experiments, lentiviral-mediated RanBPM shRNA knockdown was performed in cells cotransfected pFlag-Zta and pV5-Ub. The shRNA effectively reduced the expression of RanBPM (Fig. S1, RanBPM input). Immunoblot analysis revealed no significant change in the levels of ubiquitinated Zta in cells infected with the RanBPM shRNA virus compared to those infected with control shRNA (Fig. S1, lanes 2 and 4). Furthermore, bands of ubiquitinated Zta were absent in cells not transfected with pV5-Ub (Fig. S1, lanes 1 and 3), confirming the necessity of pV5-Ub for Zta ubiquitination detection. These results suggest that Flag-Zta ubiquitination is independent of RanBPM.

### Influence of USP11 on EBV lytic progression

**Fig 9:**
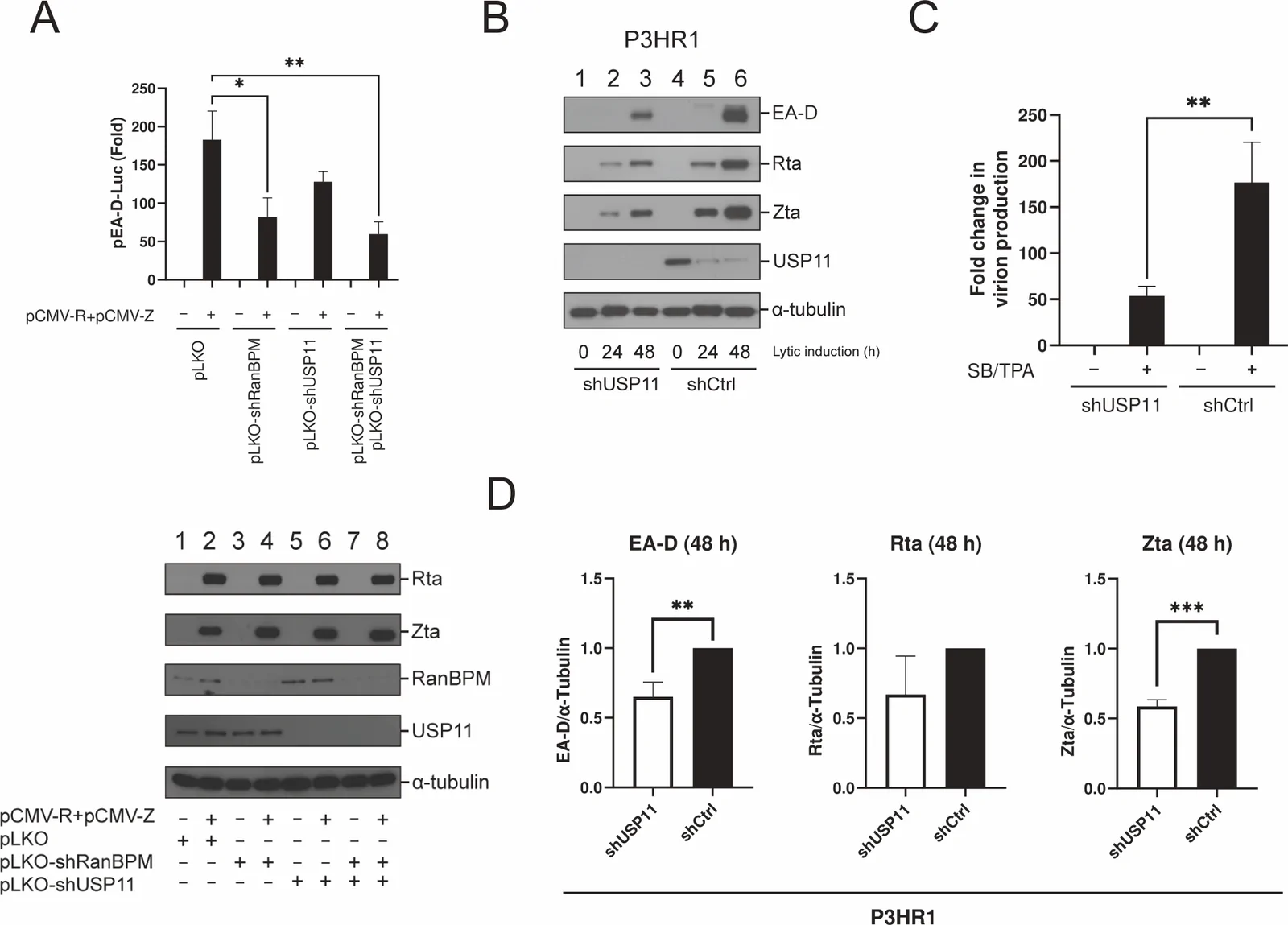
Effect of USP11 on EBV lytic development. (**A**) HEK293T cells were cotransfected with pEA-D-luc, pCMV-R, and pCMV-Z; the cells were also transfected with plasmids expressing control shRNA (shCtrl), RanBPM shRNA, USP11 shRNA, or both shRNAs, to assess the impact of RanBPM and USP11 reduction on transcription mediated by Rta and Zta. Luciferase activities were measured at 48 h after transfection. The experiment was conducted in triplicate, with each sample prepared in duplicate. Expression of Rta, Zta, RanBPM, USP11, and α-tubulin was detected by immunoblotting using the indicated antibodies. Fold activation was calculated by dividing the relative light unit (RLU) values from cells transfected with pCMV-R and pCMV-Z by the RLU values from untransfected cells. (**B**) P3HR1 cells were infected with lentivirus carrying USP11 shRNA (shUSP11; lanes 1–3) or control shRNA (shCtrl; lanes 4–6). After infection, cells were treated with sodium butyrate and TPA for 48 h. Proteins in the lysate were examined by immunoblotting using the indicated antibodies. (**C**) The lentiviral-transduced P3HR1 cells were treated with TPA and sodium butyrate for 5 days. EBV DNA from viral particles released into the culture medium was quantified by qPCR after DNA extraction. The EBV genome copy number was determined by using an EBV bacmid that had been isolated from *E. coli* as a standard. The results were obtained from three independent lentiviral infections, and each sample of qPCR was prepared in duplicate. The levels of EA-D, Rta, and Zta at 48 h in panel **B** were quantitated using ImageJ software shown in panel **D**. The results were analyzed statistically with Student’s *t*-test. *, *P* < 0.05; **, *P* < 0.01; ***, *P* < 0.001.

In our previous study, we demonstrated that Rta and Zta synergistically activate the transcription of EBV lytic genes by binding to RanBPM (18). Building upon this, we investigated whether USP11 influences this transcriptional synergy. To assess the involvement of USP11 in the Rta-Zta synergy, we cotransfected HEK293T cells with the pEA-D-Luc reporter plasmid, pCMV-R, pCMV-Z, and either RanBPM shRNA, USP11 shRNA, or both as controls. The pEA-D-Luc plasmid contains a luciferase gene transcribed from the BMRF1 promoter, which includes three ZREs, allowing for the assessment of transcriptional activation by Rta and Zta, both individually and synergistically. The results demonstrated that co-transfection of pCMV-R and pCMV-Z enhanced promoter activity by 183-fold compared to cells lacking these plasmids (Fig. 9A). Expression of RanBPM-shRNA reduced this activation to 82-fold; USP11-shRNA reduced it to 128-fold; and co-expression of both RanBPM-shRNA and USP11-shRNA decreased it to 60-fold (Fig. 9A). These findings indicate that USP11 is critical for the transcriptional synergy between Rta and Zta.

Given that Rta-Zta-mediated synergistic activation is essential for EBV lytic activation, we examined how the deubiquitination of Rta and Zta influences EBV’s lytic proliferation. Accordingly, USP11 shRNA or control shRNA was introduced into P3HR1 cells by lentiviral infection. Cells were then treated with TPA and sodium butyrate to activate the EBV lytic cycle. Results revealed that EA-D, Rta, and Zta were expressed when cells were infected with lentiviral control shRNA with the induction (Fig. 9B, lanes 5 and 6). Importantly, the amounts of EA-D, Rta, and Zta decreased at 48 h following lytic induction when cells were infected with lentiviral-based USP11 shRNA (Fig. 9B, lane 3; 9D), indicating that knocking down USP11 expression impacts the expression of lytic proteins.

Similar experiments were performed in B95-8 cells transduced with either USP11 shRNA or control shRNA. In this setting, silencing USP11 did not result in an obvious reduction in Rta, Zta, and EA-D expression following lytic induction (Fig. S2). This limited effect is likely attributable to the intrinsic properties of B95-8 cells, which are uniquely prone to EBV lytic replication and display high basal expression of lytic genes (39, 40). Such a lytic-permissive state may reduce the ability of shRNA-mediated USP11 silencing to suppress lytic activation. Consequently, the robust lytic activation in these cells may sustain Rta and Zta expression despite USP11 knockdown, resulting in only subtle changes in their levels. Finally, virion production was assessed using qPCR. The results revealed that inhibition of USP11 expression by lentiviral infection reduced the levels of virions by 70% in P3HR1 cells when the cells were treated with sodium butyrate and TPA (Fig. 9C), demonstrating that USP11 enhances EBV lytic progression. These findings suggest that USP11 plays a crucial role in the transcriptional synergy between Rta and Zta, likely through its deubiquitinating activity, which is essential for the activation of EBV lytic genes and efficient viral replication.

## DISCUSSION

Rta and Zta are transcription factors expressed by EBV during the immediate-early stage of the lytic cycle (4). These proteins can function independently or cooperate to synergistically transactivate EBV lytic genes (7, 8). Ubiquitination destabilizes both Rta and Zta (21, 33), leading us to investigate how the ubiquitination of Rta and Zta affects the transactivation of EBV lytic genes and whether deubiquitination enhances their ability to activate these genes.

In a previous study, we demonstrated that RNF4 promotes the ubiquitination of Rta (21). Given that Zta interacts with Rta to activate EBV lytic genes, we investigated whether RNF4 also serves as a ubiquitin E3 ligase for Zta. Our findings reveal that RNF4 interacts with Zta (Fig. 1) and promotes its ubiquitination (Fig. 2). Meanwhile, RNF4-mediated ubiquitination destabilizes Zta (Fig. 3), a process counteracted by USP11 (Fig. 4). Additionally, USP11 deubiquitinates Rta and Zta, enhancing their stability (Fig. 4 to 6). Our unpublished results indicate that USP11 deubiquitinates not only Rta and Zta but also two EBV minor capsid proteins, BORF1 and BDLF1, suggesting that USP11 plays a critical role in EBV lytic development. These observations suggest that host cells may utilize ubiquitination to destabilize Rta and Zta, thereby inhibiting EBV lytic development. Conversely, USP11 appears to counteract this mechanism by deubiquitinating these proteins, promoting EBV lytic progression.

RNF4 is a SUMO-targeted ubiquitin ligase (STUbL) that promotes the ubiquitination of SUMOylated Rta (21). Consequently, treatment with MG132 stabilizes SUMOylated Rta in transfection systems. However, such stabilization is not observed for SUMO-2-conjugated Zta (Fig. S3), suggesting that RNF4 does not function as an STUbL for Zta. As K12 is the only residue in Zta that is conjugated by SUMO (Fig. 2) (32), and it is also the major ubiquitination site (33), our study suggests that SUMO and ubiquitin may compete for modification at the K12 residue. Additionally, we cannot exclude the possibility that Zta is modified by K63-polyubiquitination chains for autophagic degradation (33), which could explain the ineffectiveness of MG132 in preventing the degradation of SUMO-2-Zta. Furthermore, our study found that RNF4 overexpression enhances low levels of ubiquitination on ZK12R (Fig. 2B, lane 6), suggesting that RNF4 promotes ubiquitination at several minor ubiquitination sites, including K188, K207, and K219 residues on Zta (33).

RanBPM is a protein that undergoes modification and destabilization through ubiquitination, impacting its function (34). However, USP11 interacts with RanBPM, leading to its deubiquitination and stabilization (36). In our previous study, we demonstrated that although Rta and Zta do not interact directly, they both bind to RanBPM, enabling them to activate transcription synergistically and mediating lytic activation (18). This study further reveals that USP11 interacts with both Zta and Rta (Fig. 4A and 6C). The interaction appears to remove the polyubiquitination chain formed on these two transcription factors, as reducing the expression of USP11 via USP11 shRNA significantly increased the levels of polyubiquitinated Zta and Rta, leading to their instabilities (Fig. 4B, 5 through 7). Importantly, the interaction and deubiquitination by USP11 likely occur on RanBPM, as reducing the expression of RanBPM decreases the deubiquitination by USP11 and increases the level of polyubiquitinated Rta (Fig. 8). Meanwhile, the knockdown of RanBPM expression does not seem to increase the levels of polyubiquitinated Zta (Fig. S1), suggesting that ubiquitination of Zta is independent of RanBPM.

Our study demonstrates that USP11 significantly influences the EBV lytic progression (Fig. 9). Silencing USP11 reduces the levels of an essential EBV lytic protein, EA-D, at 48 h post-induction (Fig. 9B and D). Additionally, virion production decreases in P3HR1 cells following lytic induction (Fig. 9C), indicating that USP11 plays a role in EBV lytic development. These results strongly suggest that host cells utilize RNF4 to ubiquitinate and destabilize Rta and Zta as a defense mechanism to attenuate EBV proliferation (21). Since RNF4 and TRIM5a are two E3 ligases catalyzing Rta’s ubiquitination (21, 41), it is likely that multiple E3 ligases are involved in the ubiquitination of Rta and Zta. After ubiquitinated Rta and Zta are recruited to RanBPM, they are stabilized by USP11; therefore, EBV counteracts the host defense by removing the ubiquitin chains on Rta and Zta on RanBPM, optimizing the ability of these two transcription factors to facilitate strong transactivation of EBV lytic genes, thereby favoring viral lytic progression (Fig. 9). This study underscores the critical roles of RanBPM and USP11 in EBV lytic reactivation.

This study elucidates the roles of ubiquitin E3 ligase and deubiquitinase in modulating Zta ubiquitination, a previously elusive aspect of EBV biology. Our findings demonstrate that EBV exploits USP11 to stabilize RanBPM, Rta, and Zta through deubiquitination, counteracting the ubiquitination activity promoted by RNF4. Further research into the interactions among Rta, Zta, RNF4, RanBPM, and USP11 will provide deeper insights into the mechanisms driving EBV lytic activation, with potential implications for understanding other viral reactivation processes.

## MATERIALS AND METHODS

### Cell lines

HEK293T cells were cultured in Dulbecco’s modified Eagle’s medium supplemented with 10% fetal calf serum. P3HR1 and B95-8 cells were cultured in RPMI 1640 medium supplemented with 10% fetal calf serum. These cells were treated with 3 ng/mL TPA and 3 mM sodium butyrate to activate the EBV lytic cycle and the expression of Rta and Zta (42, 43).

### Plasmids

Plasmids pCMV-R, pCMV-Z, pFlag-Rta, pHA-Ub, pV5-Ub, pFlag-Zta, pFlag-ZK12R, pFlag-RNF4, pEGFP-RNF4, pFlag-RNF4-CS1, pEGFP-RNF4-CS1, pGEX-4T1, and pCMV-3 were described previously (16–18, 21). Plasmid pTag-USP11 was kindly provided by Winston C. Y. Yu (27). Plasmid pcDNA3-Flag acts as an empty vector (Addgene). Plasmid pEGFP-USP11 was generated by inserting a PCR-amplified USP11 DNA fragment using primers USP11-HindIII-5 (5′-CCCAAGCTTATGGCGACGGTCGCAGCAAATCCAGCT-3′) and USP11-XhoI-3 (5′-CCGCTCGAGTCAATTAACATCCATGAACTCAGA), using pTag-USP11 as a template, into the HindIII-XhoI sites in pEGFP-C3. C275S and C283S substitutions in USP11, namely USP11(CS), were generated by PCR using primers USP11-CS1 (5′-GGCAACACGAGCTTCATGAACTCGGCCCTGCAGAGCCTCAGCAATG) and USP11-CS2(5′-CATTGCTGAGGCTCTGCAGGGCCGAGTTCATGAAGCTCGTGTTGCC). USP11(CS) DNA fragment was then inserted into the HindIII-XhoI sites in pEGFP-C3 to generate pEGFP-USP11(CS). Reporter plasmid pEA-D-Luc was constructed by inserting a PCR-amplified DNA fragment that contained the −332 to +20 region in BMRF1 into the HindIII–XhoI sites in pGL2-Basic (17).

### GST pulldown assay

GST, GST-USP11, and GST-RNF4 were expressed and purified from *E. coli* BL21(DE3) (pGEX-4T1), *E. coli* BL21(DE3) (pGEX-USP11), and *E. coli* BL21(DE3) (pGEX-RNF4), respectively, according to the methods described earlier (44). GST-pulldown assay was conducted as described previously (44).

### Immunoprecipitation

HEK293T cells were transfected with plasmids using Turbofect reagent (Thermo Fisher Scientific). At 24 or 48 h after transfection, a lysate was prepared using RIPA buffer (16), sonicated, and centrifuged at 13,800 × *g* for 10 min according to a method described elsewhere (16). An anti-Rta monoclonal antibody (Argene) or an anti-RNF4 monoclonal antibody (21) was mixed with the supernatant at 4°C for 1 h. Protein-A-agarose or protein-G-agarose beads were added to the lysate and incubated at 4°C for 1 h. The beads were collected by centrifugation and washed three times with PBS. Proteins binding to the beads were eluted with electrophoresis sample buffer and analyzed by immunoblotting (44).

### Denature immunoprecipitation assay

To detect ubiquitinated proteins, transfected cells were harvested and washed with PBS containing 10 mM N-ethylmaleimide. Cells were then suspended in 100 µL denatured lysis buffer (21), containing 1% SDS, and then incubated at 95°C for 10 min. The lysate was then diluted with 900 µL wash buffer for subsequent immunoprecipitation and immunoblotting (21).

### Analysis of protein stability

HEK293T cells were cotransfected with pCMV-R, pcDNA-Zta-myc-His, and pEGFP-USP11 or USP11 shRNA. At 40 h after transfection, cells were treated with 200 µg/mL CHX. Cells were collected at different time points after the treatment. Rta, Zta, and α-tubulin in the lysate were detected by immunoblot analysis. The intensity of the protein bands was determined using ImageJ software to assess the rate of protein decay.

### Knockdown of USP11 and RanBPM expression using shRNAs

USP11 shRNAs (target sequence: 5′-CCGTGACTACAACAACTCCTA) and RanBPM shRNAs (target sequences: 5′-GCTCTAAACATGACCACGAAA and 5′-GCATTCAGTCTACTAGCATAT) and plasmids including pVSV-G, pCMVDR8.91, and pLKO-shRNA were purchased from the National RNAi Core Facility, Genomic Center, Academia Sinica, Taipei, Taiwan. Knockdown of USP11 expression in P3HR1 cells and B95-8 cells was performed using lentiviral infection according to the method described elsewhere (21).

### Transient transfection and luciferase assay

HEK293T cells (1 × 10^4^) cells were transfected with 0.2 µg pEA-D-luc, pCMV-R, pCMV-Z, or the control vector pCMV-3 in the presence of RanBPM shRNA, USP11 shRNAs, or control shRNA using Turbofect reagent (Fermentas). To assess promoter activities, the cells were harvested, washed with PBS, and then lysed in lysis buffer (21). Luciferase activity was measured using a luminometer (Orion II; Berthod, Bad Wildbad, Germany) (21). Each transfection experiment was performed at least three times, with each sample prepared in duplicate.

### Quantification of the EBV genome

P3HR1 cells were treated with TPA and sodium butyrate to activate the lytic cycle. At day 5 after induction, virions released into the culture medium were collected by ultracentrifugation at 25,000 × *g* for 2 h. qPCR was performed according to the method as described (21).

## SUPPLEMENTAL MATERIAL

Supplemental figures spectrum.04158-25-s0001.docx Fig. S1–S3.

ASM does not own the copyrights to Supplemental Material that may be linked to, or accessed through, an article. The authors have granted ASM a non-exclusive, world-wide license to publish the Supplemental Material files. Please contact the corresponding author directly for reuse.
