# Supplementary material for: Impact of deubiquitination of Epstein-Barr virus Rta and Zta on lytic development
Source: Microbiol Spectr. 2026 May 21;14(7):e04158-25. doi: 10.1128/spectrum.04158-25 (PMC13339912; doi:10.1128/spectrum.04158-25)
Supplement: Supplemental figures — Fig. S1–S3. [file spectrum.04158-25-s0001.docx]

**Supplemental materials**

**
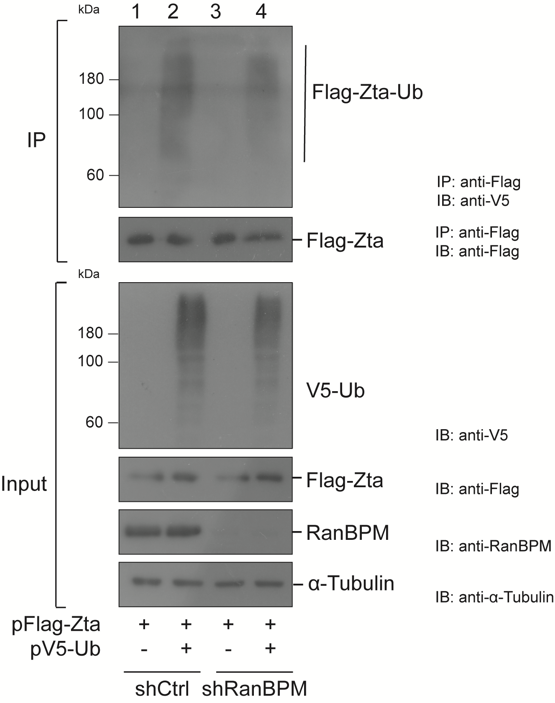
**

**Fig. S1. Ubiquitination of Zta is independent of RanBPM.**

HEK293T cells were infected with lentivirus expressing either RanBPM shRNA (lanes 3, 4) or control shRNA (lanes 1, 2) and then cotransfected with plasmids expressing Flag-Zta and pV5-Ub. At 48 h after transfection, cells were treated with 5 μM MG132 for 8 h to inhibit proteasomal degradation of Ub-Zta. Proteins in the lysate were then immunoprecipitated with an anti-Flag antibody and detected with an anti-V5 antibody. Proteins in the lysate were also immunoblotted with anti-V5, anti-Zta, anti-RanBPM, and anti-α-tubulin antibodies. Input lanes were loaded with 5% of the lysate for the detection by anti-V5, anti-Zta antibodies; 1% of the lysate for the detection by an anti-α-tubulin antibody.

**
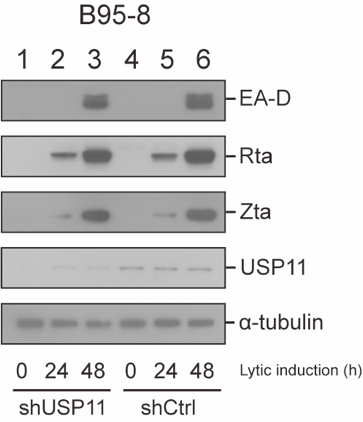
**

**Fig. S2. Influence of USP11 on EBV lytic cycle in B95-8 cells.** B95-8 cells were infected by lentivirus carrying USP11 shRNA (shUSP11) (lanes 1-3) or control shRNA (shCtrl) (lanes 4-6). After infection, cells were treated with sodium butyrate and TPA for 48 h. Proteins in the lysate were examined by immunoblotting using indicated antibodies.


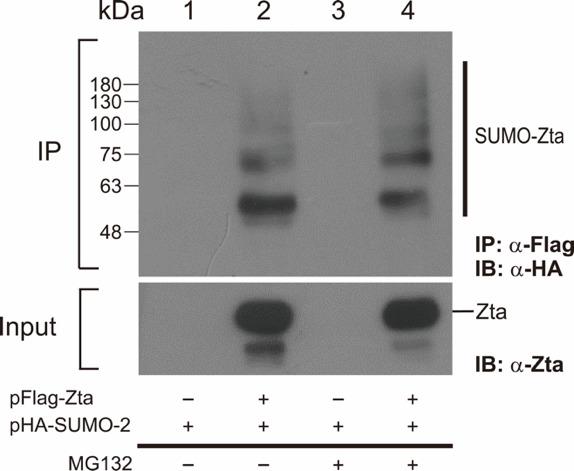


**Fig. S3. SUMOylation of Zta is not required for the ubiquitination of Zta.** HEK293T cells were cotransfected with pFlag-Zta and pHA-SUMO-2. At 24 hours after transfection, cells were treated with dimethyl sulfoxide (DMSO) or 2.5 μM MG132 for another 12 hours. SUMOylated Zta (SUMO-Zta) in the lysate were immunoprecipitated (IP) using an anti-Flag antibody, and proteins bound to anti-Flag-M2 agarose beads were analyzed by immunoblotting (IB) using an anti-HA antibody.
